# Human African Trypanosomiasis Diagnosis in First-Line Health Services of Endemic Countries, a Systematic Review

**DOI:** 10.1371/journal.pntd.0001919

**Published:** 2012-11-29

**Authors:** Patrick Mitashi, Epco Hasker, Veerle Lejon, Victor Kande, Jean-Jacques Muyembe, Pascal Lutumba, Marleen Boelaert

**Affiliations:** 1 Department of Tropical Medicine, Parasitology Service, Faculty of Medicine, Kinshasa University, Kinshasa, Democratic Republic of Congo; 2 Department of Public Health, Group Epidemiology and Disease Control, Institute of Tropical Medicine, Antwerpen, Belgium; 3 Department of Biomedical Sciences, Institute of Tropical Medicine, Antwerpen, Belgium; 4 Programme National de Lutte contre la Trypanosomiase Humaine Africaine, Kinshasa, Kinshasa, Democratic Republic of Congo; 5 Institut National de Recherche Biomédicale, Avenue de la Démocratie, Kinshasa, Democratic Republic of Congo; University of Pittsburgh, United States of America

## Abstract

While the incidence of Human African Trypanosomiasis (HAT) is decreasing, the control approach is shifting from active population screening by mobile teams to passive case detection in primary care centers. We conducted a systematic review of the literature between 1970 and 2011 to assess which diagnostic tools are most suitable for use in first-line health facilities in endemic countries. Our search retrieved 16 different screening and confirmation tests for HAT. The thermostable format of the Card Agglutination Test for Trypanosomiasis (CATT test) was the most appropriate screening test. Lateral flow antibody detection tests could become alternative screening tests in the near future. Confirmation of HAT diagnosis still depends on visualizing the parasite in direct microscopy. All other currently available confirmation tests are either technically too demanding and/or lack sensitivity and thus rather inappropriate for use at health center level. Novel applications of molecular tests may have potential for use at district hospital level.

## Author Summary

We conducted a systematic review of the English and French language literature indexed in PubMed between January 1970 and December 2011. Our objective was to identify which diagnostic tests are most suitable for diagnosis of Human African Trypanosomiasis (HAT) in first line health services in endemic countries. Tests were rated according to the “ASSURED” criteria, which put emphasis not only on diagnostic accuracy but also on user-friendliness and feasibility under field conditions. Diagnosis of HAT is a two-stage process in which a screening test is followed by a diagnostic confirmation test. Our search retrieved 16 different screening and diagnostic confirmation methods. The thermostable format of the CATT test came out as the most suitable screening test at health center level. It might be replaced by a lateral flow test that is currently being validated. Diagnostic confirmation tests currently available are cumbersome, technically demanding and have inadequate sensitivity; they rely on visualizing the parasite by microscopy. Without specific training and supervision, none of these tests can readily be used at health center level. Novel applications of molecular tests have the potential to replace the current diagnostic confirmation tests at district hospital level but they first need to be validated.

## Introduction

Human African Trypanosomiasis (HAT) is a parasitic disease transmitted by an insect vector, the tsetse fly. The disease is endemic in rural areas of sub-Saharan Africa. More than 90% of cases are due to infection with *Trypanosoma brucei* (*T.b.*) *gambiense*, causing West-African HAT [1] which is assumed to be an anthroponosis and is the subject of the current review. Control of West-African HAT (henceforward abbreviated as HAT) is based on two strategies: i) case detection followed by treatment of confirmed cases and ii) vector control [1]. Until recently the main control approach was based on ‘active screening’, usually done by mobile teams that move from village to village to examine entire village populations in HAT endemic areas. This mode is contrasted with ‘passive screening for HAT’- organized from fixed health structures among patients self-presenting for consultation, either at their own initiative or after referral by other health care providers; passive screening is sometimes extended to the relatives accompanying these patients [2]. Both active and passive screening approaches for HAT follow a two-step diagnostic procedure. Since HAT has no distinctive early signs and symptoms, the first step is a serological test which detects trypanosome-specific antibodies. Those serologically positive are considered ‘suspect’ and are subjected to parasitological confirmation tests [3], [4]. The most commonly used serological test in the field is the Card Agglutination Test for Trypanosomiasis (CATT)/*T.b. gambiense* [5]. As a rule patients are treated for HAT only if trypanosomes have been detected in their body fluids, although in areas of high endemicity high titers in serology are also used by some as a criterion for initiating treatment [6].

If properly managed, annual rounds of active screening for HAT during three consecutive years can significantly reduce transmission of HAT in a community [2], [7]. However, from historic evidence and carefully documented case studies we know that HAT transmission is very hard to eradicate. After a number of years of low or zero cases, HAT inevitably comes back if no further control measures are implemented [1]. Some sustained surveillance is therefore needed [7]. However, population screening for HAT requires significant and sustainable funding. When the prevalence is reduced, costs per detected case increase and governments and donors become reluctant to continue funding [8]. Moreover the population does no longer consider HAT a threat and becomes reluctant to participate in the time consuming screening exercise [9]. Therefore HAT control programs nowadays tend to integrate HAT control activities into general health services [8]. These services are typically organized along the model of the district health system with two echelons, the health center as first line and the district hospital as second line. Health centers are typically run by mid-level health workers and as a rule lack resources such as sophisticated diagnostic equipment and electricity [10]. More importantly, as HAT typically occurs in very remote and isolated areas, the health centers in those places are equally fragile, understaffed, and ill-equipped. In the coming decade it will be crucial how those health services will deal with the HAT problem. Can they adequately diagnose and treat patients with HAT who spontaneously present to their clinics? We performed a systematic literature review of all diagnostic and/or screening tests for HAT *T.b. gambiense*; our objective was to identify which tests can be safely recommended for use in primary care health centers in endemic areas.

## Materials and Methods

### Context

The focus of our review was to identify diagnostic and/or screening tests for HAT that can be used at the level of a primary care health center in endemic areas. Though the situation may differ between and within countries, for the purpose of this review we assume that those rural health centres are typically run by mid-level health workers, lack electricity supply, and have no regular cold chain.

### Literature search

We conducted a literature search in PubMed to identify relevant original articles related to diagnostic and screening tests for *T.b. gambiense* HAT, published between January 1970 and December 2011. We used the following search strategy: “Trypanosomiasis, African/diagnosis”[Mesh] AND (Humans [restriction] language: English OR French AND (“1970/01/01”[PDat] : “2011/12/31”[PDat])). Titles and abstracts were screened using the following inclusion criteria:

- original articles, and
- articles on screening or diagnostic tests for *T.b.gambiense* HAT, and
- articles providing estimates of sensitivity and specificity and/or information on feasibility of those tests.

The full paper was retrieved for all abstracts meeting inclusion criteria and those papers were further screened by PM. We excluded all studies evaluating solely diagnostic tests for *Trypanosoma brucei rhodesiense* or solely tests used to determine the stage of HAT. We also excluded all tests requiring a lumbar puncture, as this technique is not feasible at health centre level. All diagnostic and screening tests which were not currently in production and available for use in the field were also excluded.

We screened the references of all articles retained to identify further articles meeting our inclusion criteria.

### Evaluation criteria

We used the conceptual framework for quality assurance of diagnostic tests developed by Peeling and colleagues [10]. The sexually transmitted diseases diagnostics initiative (STDI) has summarised characteristics of the ideal diagnostic test for remote field settings [10], [11], and coined these as the ASSURED criteria. The test must be Affordable, Sensitive (few false-negatives), Specific (few false-positives), User-friendly (simple to perform and requiring minimal training), Robust and rapid (can be stored at room temperature and results available in <30 min), Equipment-free or requiring minimal equipment that can be solar-powered and Deliverable to those who need them. Each test was judged against the individual criteria of this framework.

## Results

**Figure 1:**
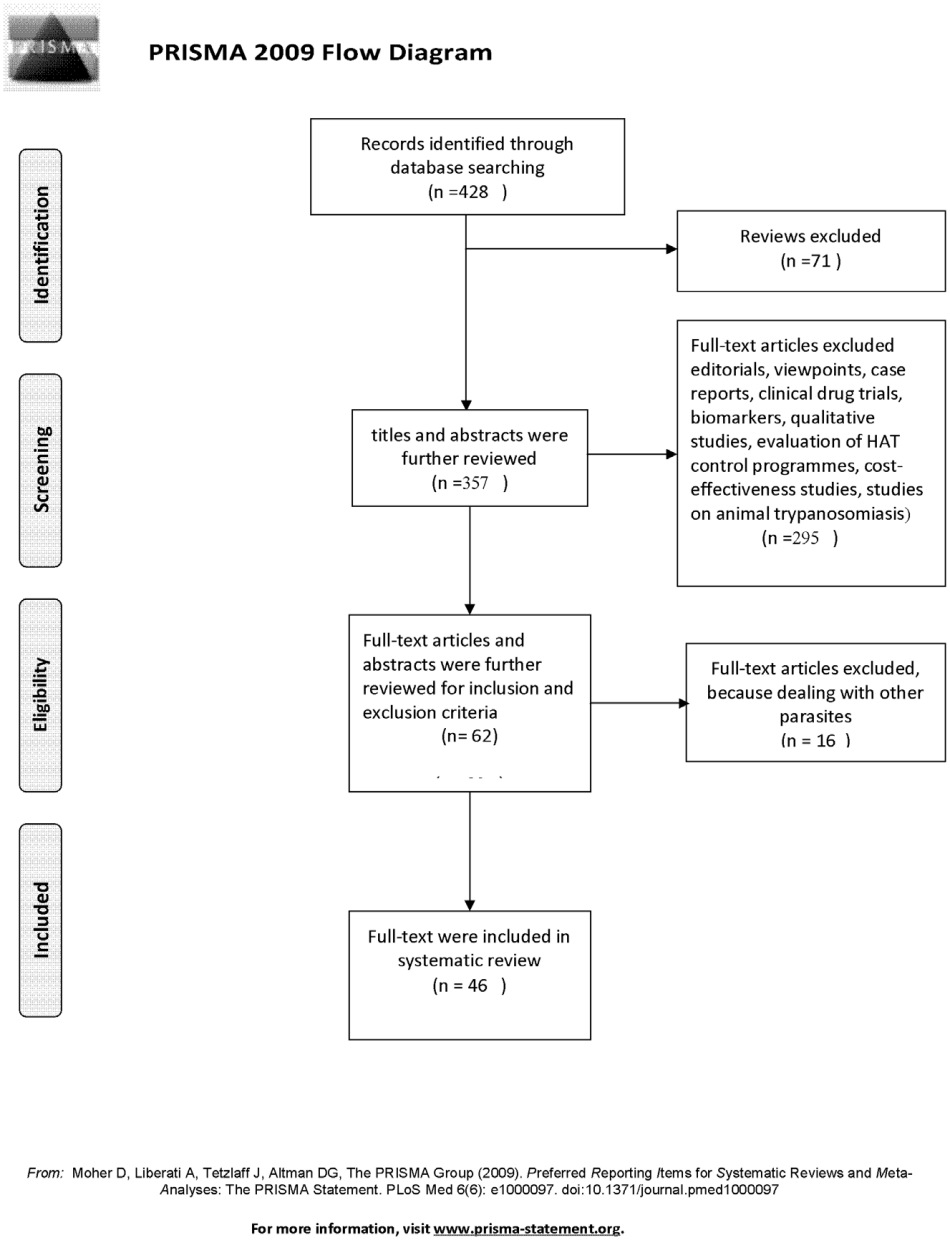
Flow diagram for study selection.

Our literature search identified 428 potentially relevant studies, of which 71 were excluded because they were reviews. Out of the remaining 357 studies, 295 were excluded based on the abstracts. Of 62 studies remaining, 16 were excluded because they were dealing with other parasites; the remaining 46 articles were included in the review (see figure 1).

The articles retained for analysis reported on five serological antibody detection tests: CATT, Latex*/T.b. gambiense*, Immune trypanolysis test, ELISA and IFAT. CATT is available in different formats which were evaluated separately; these include the CATT on undiluted blood, the CATT on sample dilution, the CATT-D10, and the CATT on filter paper. Seven different parasite detection methods were identified: the Wet Blood Film (WBF), Thick Blood Film (TBF), lymph node aspirate (LNA), mini-Anion Exchange Centrifugation Technique (mAECT), mAECT of the buffy coat (mAECT-bc), the micro-haematocrit centrifugation technique (mHCT), and the Quantitative Buffy Coat (QBC). Finally there were several studies evaluating molecular methods based on amplification of parasite DNA and/or RNA with or without a thermocycler, these include the Polymerase chain reaction (PCR), oligochromatography-PCR, real-time nucleic sequence based amplification (NASBA) and Loop-mediated isothermal amplification (LAMP), of which the latter is still under development. None of the above tests were proprietary diagnostic devices; all were either based on an in-house procedure or a non-commercial production and supply process. We will now describe each of the tests identified and assess their feasibility for use at health center level according to the criteria of the ASSURED framework.

### Serological antibody detection tests

#### CATT

The CATT is a screening test for HAT. It is a direct agglutination test based on a freeze-dried purified, formaldehyde-fixed and Coomassie-blue-stained bloodstream-form of *T. b. gambiense* variable antigen type LiTat 1.3. Three CATT formats are available: the classical CATT test on whole blood [5], the CATT on filter paper [12]–[17] and the CATT-D10 [18]. The CATT is a fast and simple agglutination assay for detection of *T. b. gambiense*-specific antibodies in the blood, plasma, or serum of HAT patients. The reagent is mixed with the sample (blood, serum, plasma) and shaken for 5 min on a rotator at 60 rotations per minute; the result is visible to the naked eye. Up to ten patients can be tested at the same time and hundreds of individuals can be screened daily. The reagent has to be kept refrigerated. The rotator works on 12 V DC and is usually operated on a car battery; it can also be operated on eight 1.5 V battery cells. Reported sensitivity of CATT on undiluted blood varies from 68·8% to 100% [13], [15]–[17], [19]–[23], reported specificity ranges between 83·5% and 99·3% [13], [15], [17], [19]–[25] and reproducibility is good (Kappa coefficient = 0·84) [18]. The CATT test fulfils each of the ASSURED criteria except robustness since it is not thermostable (table 1). Moreover once a 50-unit vial is reconstituted it needs to be used the same day.

**Table 1:** Evaluation of feasibility of all methods according to ASSURED criteria.

	Affordable(Euro)	Sensitivity(%)	Specificity(%)	User-friendly	Rapid(minutes)	Robust	Equipment-free	Deliverable
Tests								
CATT	0•46	68.8–100	83•5–99•3	Yes	<30	No	Yes	Yes
CATT dilution	1	78•8	58•5–99•5	No	<30	No	No	Yes
MicroCATT	0•8	91–92•7	93•7–100	No	<30	No	No	Yes
CATT-D10	1•5–2	NR	NR	Yes	<30	Yes	Yes	Yes
Latex	0•8	67•9–100	96•1–99•2	No	<30	No	No	Yes
IFAT	5–7	75•6–99•2	99•4–100	No	>120	No	No	No
ELISA	0•62	96•3–100	94•7–100	No	>120	No	No	No
Immune trypanolysis test	>5	97•2–100	100	No	>120	No	No	No
WBF	0•21	3•9–54•2	1†	Yes	16	n.a	Yes	No
TBF	0•54	25•9–100	1†	Yes	47	Yes	Yes	No
LNA	0•19	18•8–63•6	1†	Yes	16	n.a	Yes	No
mHCT	0•76	44•3–93•0	1†	No	18	Yes	No	No
QBC	3	100	1†	No	<30	Yes	No	Yes
mAECT	3	75•3–90•9	1†	No	∼30	Yes	No	Yes
mAECT-bc	3	96•5	1†	No	>30	Yes	No	Yes
PCR	2•6–4•8	70•0–100	71•4–100	No	120–240	No	No	No
PCR-OC	2•6–4•8	82•4–100	99•2–100	No	120–240	No	No	No
NASBA	5•20	70•0	100	No	120	No	No	No
NASBA-OC	4•0–4•5	73•0–97•1	99•2–100	No	90	No	No	No
LAMP	5	75•0	100	No	30–60	No	No	No

1† = specificity of these techniques is assumed to be 100%.

**CATT on sample dilutions** can be performed on blood, plasma and serum. It is based on serial dilutions, starting from 25 µl blood, plasma or serum dilution, mixed with 45 µl of CATT reagent. The reading occurs after 5 min of mixing. The sensitivity of a 1∶8 dilution performed with plasma or serum is estimated at 78·8% [26] and its specificity ranges from 58·5% to 99·5% [13], [26]. CATT titration is used to achieve an increase in specificity when compared to CATT on whole blood [27]. Persons with an end titer ≥1∶8 in whom parasitological confirmation tests remain negative are called ‘serological HAT suspects’ and may be treated in certain circumstances [6]. The preparation of sample dilutions requires trained personnel and some extra equipment in addition to the standard requirements for the classical CATT tests, such as micro-titer plates, micropipettes and extra buffer volumes. Referring to the ASSURED criteria the CATT test on sample dilutions has the same limitations as the classical CATT test, apart from that it requires additional equipment and technical skills (table 1).

CATT on filter paper or **micro-CATT** is an application of the CATT test using eluates from dried capillary blood samples on filter paper. Reagents used are the same as those used in the classical CATT test. The impregnated-blood filter paper can be collected by community health workers without special training and thus may make it possible to screen large populations at relatively low cost [28]. The reported sensitivity of the CATT on filter paper varies from 91% to 92·7% [12]–[17], specificity ranges from 93·7% to 100% [13]–[17], [29]. CATT on filter paper is generally less sensitive than the classic CATT test using samples of fresh capillary blood [17]. In one study CATT on filter paper lost its sensitivity rapidly when the filter papers were stored at ambient temperature [17]. Reproducibility of CATT on filter paper is excellent (Kappa = 0·84) [12], [14]. The micro-CATT does not meet the ASSURED criteria of robustness, being equipment free and user- friendly (table 1).

A more recently developed format, the **CATT-D10**, is constituted with the same antigen as the classic CATT test but with a different lyophilisation medium. It is produced in vials of 10 dosages (instead of 50 dose vials for the classical CATT) and remains stable when stored dry at ambient temperature. In principle this allows for the use of CATT as a screening test at health center level (table 1). Reproducibility between CATT-D10 and classical CATT is excellent (Kappa = 0·83) sensitivity and specificity are in the same range as the classical CATT. The CATT-D10 has all the advantages of the classical CATT and has higher thermostability, thus fulfils each of the ASSURED criteria (table 1).

The **Latex** ***/T.b. gambiense*** test [30] is based on indirect agglutination of specific antibodies with antigens coupled to the surface of latex beads. The antigen consists of a mixture of variable surface glycoproteins of *T.b. gambiense* variable antigen type 1.3, 1.5 and 1.6. Blood, serum or plasma dilutions are prepared in micro titer plates. Presence of antibodies in the blood is revealed by a white macroscopic agglutination after rocking the card on a horizontal rotator at 70 rpm for 5 min. Sensitivity is estimated at 67·9–100% [17], [22], [30] while specificity is estimated at 96·1%–99·2% [13], [17], [22], [25], [30]. As for the CATT test on dilutions, the Latex test requires additional materials (pipettes, microtiter plates), cold chain, and trained personnel [25]. These requirements make it unlikely to ever become a routine test at health center level. With reference to the ASSURED criteria, the Latex test has a number of limitations (lack of user friendliness, lack of robustness, and requirement of equipment) that limits its potential for utilization in first line facilities (table 1).

#### IFAT

Developed in the 1970s, IFAT uses IgG-specific fluorescent conjugate for detection of *T.b. gambiense*-specific IgG in test sera, dried blood samples on filter paper and cerebrospinal fluid. *T.b. gambiense* or *T.b. brucei* are maintained by serial inoculation in mice; parasitized blood from mice is used as antigen in different protocols [31]. This antigen is usually prepared from parasitized blood smears which contain between 5 and 50 parasites per field, fixed in acetone, and requires low temperature (−15°C) storage. A positive reaction is visualized by microscopy with ultraviolet light. Reported sensitivity varies from 75·6% to 99·2%, specificity from 99·4% to 100% [31]–[35]. The need of large volumes of buffer for washing steps and the need of a fluorescent microscope restrict the use of IFAT to better equipped facilities. The latter could be overcome through the use of an LED microscope; however this would still require further validation studies. The availability of standardized and stabilised antigen for *T.b. gambiense* at low cost has greatly improved the reliability of the test. However, comparison of IFAT results is difficult due to the variations between prepared blood smears and the subjectivity of microscopic interpretation. IFAT does not meet a number of ASSURED criteria: affordability, user-friendliness, rapidity, robustness, being equipment-free and being deliverable; precluding its use in first line facilities (table 1).

#### ELISA methods

ELISA is an antibody detection test that uses the same antigens as the Latex/*T.b.gambiense* (LiTat 1.3+1.5+1.6) but fixed in an ELISA plate [14]. Test sample may be serum, plasma or saliva, either fresh or on filter paper. Reported sensitivity with different kinds of samples ranges from 82·8% to 100%, specificity from 94·7% to 100% [13], [14], [20], [36], [37]. Performing ELISA tests is time-consuming, technically demanding and expensive; moreover ELISA tests are not an individual format tests but are performed in batches of at least 50 samples. These limitations preclude their use in first line health facilities. The need of sophisticated equipment, electricity, trained personnel and large volumes of purified water remain serious drawbacks for wide spread application of the tests. For surveillance purposes ELISA could be used on eluates of dried blood from filter papers in a reference laboratory, provided the optimal cut off has been determined [14]. ELISA does fail however on several of the ASSURED criteria, in particular due the fact that it requires sophisticated equipment and skilled staff (table 1).

#### Immune trypanolysis test

The antigen of the immune trypanolysis test is derived from cloned populations of live trypanosomes of a determined variable antigenic type (VAT). Live trypanosomes are incubated at 37°C with a diluted serum sample of the patient and serum from a guinea pig, rich in complement. If specific antibodies are present in the serum of the patient, they will bind to the corresponding variant surface glycoprotein. The immune complex thus activated, causes the lysis of trypanosomes. The preparation is examined under a microscope; the absence of motile trypanosomes is a positive reaction [38]. The trypanolysis is not a routine test; it can be performed only in a specialized laboratory because of the need of maintaining cloned trypanosomes. In addition, the manipulation of live *T.b. gambiense* trypanosomes poses a high infection risk to the laboratory personnel. Reported sensitivity and specificity of trypanolysis are estimated at 97·2 to 100% [26], [39], [40] and 100% respectively [38]. This test can only be performed in well-equipped reference laboratories because of requirement of an in *vivo* culture of live, cloned human-infective trypanosomes and because it requires highly trained personnel. The test can be applied on blood samples on filter paper and could thus be used in epidemiological surveillance of HAT. Further validation is still required though. Though immune trypanolysis does meet the ASSURED criteria of being sensitive and specific, it is not robust, not rapid, not equipment-free and not deliverable (table 1).

### Parasite detection methods

The principle behind these methods is visualization of the causative parasite, *T.b. gambiense*. Most parasitological techniques, except the thick blood film, detect live trypanosomes and should be performed without delay. Specificity of these techniques is assumed to be 100%. Though their sensitivity is far from satisfactory, in the absence of more sensitive tests that are equally specific, they are considered to be the gold standard. For this reason in the section below we will only report on sensitivity of the techniques, unless other estimates of specificity were available in the literature.

#### Trypanosome detection in the blood and lymph

In **wet blood films**, 5 to 10 µl of finger prick blood is placed on a slide and examined microscopically (magnification, ×400) under a cover slip. Trypanosomes can be seen moving between the erythrocytes (the movement of the surrounding erythrocytes often attracts attention). It requires no reagent and microscopic reading is facilitated by the mobility of the trypanosome. Reported sensitivity is between 3·9% and 54·2% [26], [41], [42] (table 2). This method is still frequently used in first line health facilities because it does meet the ASSURED criteria of affordability, rapidity and simplicity; however it fails on the key criterion of high sensitivity (table 1).

**Table 2:** Summary of published sensitivity estimates of parasite detection methods for HAT.

Authors	Participant countries	Test	Number of participants	Sensitivity(CI 95%)
Henry et al[^41^]	DRC	WBF	96	54•2 [43•8–64•3]
Miezan et al[^42^]	IC	WBF	58	22•4 [11•9–37•7]
Lutumba et al[^26^]	DRC	WBF	154	3•9[1•6–8•7]
Henry et al[^41^]	Zaïre(DRC)	TBF	96	82•3 [72•9–89•1]
Miezan et al[^42^]	IC	TBF	58	34•5 [22•8–48•2]
Lutumba et al[^26^]	DRC	TBF	154	25•9 [19•3–33•7]
Bailey et al[^46^]	Uganda	TBF	30	93•3 [76•5–98•8]
Truc et al[^49^]	IC	TBF	11	100 [67•9–99•2]
Henry et al[^41^]	DRC	LNA	96	51•6 [41•2–61•8]
Nantulya et al[^48^]	Uganda/IC	LNA	77	63•6 [51•8–74•0]
Miezan et al[^42^]	IC	LNA	58	58•6 [44•9–71•1]
Lutumba et al[^26^]	DRC	LNA	154	18•8 [13•0–26•3]
Bailey et al[^46^]	Uganda	LNA	30	63•3 [43•9–79•4]
Truc et al[^49^]	IC	LNA	11	63•6 [31•6–87•6]
Duvallet et al[^47^]	IC	LNA	95	51•6[41•2–61•9]
Duvallet et al[^47^]	IC	mHCT	95	86•3[77•4–92 •2]
Nantulya et al[^48^]	Uganda,IC	mHCT	77	44•2[33•0–55•9]
Miezan et al[^42^]	IC	mHCT	58	48•3[35•1– 61•7]
Lutumba et al[^26^]	DRC	mHCT	154	56•5[48•3–64•4]
Truc et al[^49^]	IC	mHCT	11	54•5[24•5–81•8]
Mcnamara et al[^50^]	Uganda	mHCT	19	94•7[71•8–99•7]
Bailey et al[^46^]	Uganda	QBC	30	100[85•9–99•70]
Truc et al[^49^]	IC	QBC	11	100[67•8–99•2]
Mcnamara et al[^50^]	Uganda	QBC	19	100[79•1–99•5]
Truc et al[^49^]	IC	mAECT	11	90•9[57•1–99•5]
Nantulya et al[^48^]	IC	mAECT	77	85•7[75•4–92•3]
Miezan et al[^42^]	IC	mAECT	58	84•5[72•1–92•2]
Lutumba et al[^26^]	DRC	mAECT	154	75•3[67•6–81•7]
Camara et al[^52^]	G-E	mAECT-bc	57	96•5[86•8–99•4]

IC = Ivory Coast; DRC = Democratic Republic of Congo; G-E = Guinée Equatoriale.

**Thick blood films (TBF),** a small drop of blood (∼10–20 µl) is placed on a glass slide and spread to approximately 4 times its original surface. After extensive drying, the slides can be stained and read under a microscope at 1000 times magnification. The Giemsa-based staining procedure for thick film preparations takes approximately 30 minutes. Electricity it not necessarily required, no centrifuge is used. Identification of trypanosomes, which are frequently deformed in this preparation, can be cumbersome and requires considerable expertise and training. Reliability of results is dependent upon the skills of the laboratory technician and the quality of reagents [43], [44]. The sensitivity of the TBF in different studies was estimated at 25·9 to 100% [26], [41], [42] (table 2). TBF fulfils some ASSURED criteria (user-friendliness, being equipment-free, and being deliverable) but it is not sensitive enough to be relied upon as diagnosis method in first-line health facilities (table 1).

For examination of the **lymph node aspirate** (LNA), a posterior enlarged cervical lymph node is punctured with a needle and the fluid examined at a magnification of 400× [43]. This test requires no reagents and no electricity. During the routine work of a mobile team, many people may be examined in one day. In the most HAT endemic country, the DRC, more cases are confirmed by the LNA than by any other method [45]. Although LNA is test most frequently used in the field because of simplicity and low cost; it should be preceded by a screening test because enlarged cervical lymph nodes are not specific for HAT but may also be signs of several other tropical diseases such as leishmaniasis, tuberculosis, malaria, toxoplasmosis and HIV infection. The sensitivity is estimated from 18·8% to 63·6% [26], [41], [46]–[49] (table 2). Though LNA meets most of the ASSURED criteria its lack of sensitivity is a major constraint (table 1).

**Mini haematocrit centrifugation technique (mHCT)** allows collection of 50–70 µl of blood in a capillary tube. After centrifugation, live trypanosomes are concentrated in the white blood cell zone between the plasma and the erythrocytes [43]. When using four to eight capillary tubes, reported sensitivity is 44·3% to 93·0% [26], [42], [47]–[50] (table 2). The mHCT is simpler than many other tests but requires a haematocrit centrifuge, electricity, and trained laboratory technicians. Thus it fails the ASSURED criteria of user-friendliness and being equipment-free (table 1).

**Quantitative Buffy Coat (QBC)** uses acridine orange and ethylene diamine tetra acetic (EDTA) coated in special capillary tubes. After high-speed centrifugation of the blood in capillary tubes provided with a floating cylinder, live trypanosomes can be visualised by fluorescent microscopy. The technique requires specific equipment (QBC centrifuge and tubes, fluorescent light source), electricity and extensive training of laboratory technicians. In initial studies QBC showed excellent sensitivity (100%) but the technique was never evaluated on large scale [46], [49], [50] (table 2). QBC mainly fails the ASSURED criteria of being affordable and equipment-free (table 1).

In the **mini Anion Exchange Centrifugation Technique (mAECT)**, live trypanosomes are separated from the blood by anion chromatography and then concentrated at the bottom of a collecting tube by low-speed centrifugation and visualized by microscopy (×100 magnification) [51]. mAECT uses 350 µl of blood, resulting in high sensitivity, but the manipulations are quite tedious. The sensitivity of mAECT is estimated at 75·3%–90·9% [26], [42], [48], [49] (table 2). This test is not user-friendly, is not equipment-free may and is relatively costly. Another format of mAECT has recently been developed: mAECT-bc (mini Anion Exchange Centrifugation-buffy coat). In this test, 350 µl of buffy coat taken after centrifugation (5 min at 1500 g) of the 5 ml of heparinised blood are processed on mAECT tests columns. A preliminary evaluation of mAECT-bc showed higher sensitivity (96·5%) [52]. This technique still needs to be evaluated on a large-scale before it can be used as routine tool for HAT diagnosis (table 1). However even if its diagnostic accuracy is confirmed it will still not meet the ASSURED criteria of being ‘user friendly’ and ‘equipment free’.

### Molecular methods

**Polymerase chain reaction (PCR)** assays detecting parasite DNA constitute the main approach for molecular detection of trypanosomes. Several PCR formats are available mostly based on primers targeting the 177 bp satellite DNA. These primers permit detection of the subgenus *Trypanozoon* (*T brucei s.l*, *Trypanosoma evansi* and *Trypanosoma equiperdum*) [53]. They do not discriminate between the species pathogenic to humans, i.e. *T.b. gambiense* and *T.b. rhodesiense*. The primers based on the gene encoding *T. brucei gambiense*-specific glycoprotein (TgsGP) for *T. brucei gambiense* and that encoding the serum-resistance-associated protein (SRA) for *T.b. rhodesiense* do discriminate between the two subspecies associated with HAT [54] and differentiate from the non-human pathogenic (sub)species as well.

Among the distinct PCR methods available are: conventional PCR [54]–[58], real-time PCR [59] and low-tech PCR approaches such as loop-mediated isothermal amplification (LAMP) [60]–[63], real-time nucleic sequence-based amplification (NASBA) [64]–[67], and oligochromatography-PCR [68]. Test samples, which are stabilized in buffer or absorbed on filter paper, may be whole blood, buffy coat, lymph node aspirate or cerebrospinal fluid. Unexplained false-negative results were observed in parasitologically confirmed cases [69], [70]. PCR is intrinsically susceptible to minute quantities of contaminating DNA or inhibition factors. Although DNA-based methods have shown excellent sensitivity (70·0%–100%) and specificity (91·8%–100%) (table 3), the introduction of these methods in daily laboratory practice is still uncommon especially in rural HAT endemic regions. The lack of standardisation and need for quality control are major concerns for PCR assays with many published reports involving a multitude of gene targets, protocols and applications. There is thus an urgent need for standardisation and optimisation of these techniques, which remain expensive and sophisticated and are therefore restricted to a few research centres in most HAT endemic countries. Clearly these tests do not meet the ASSURED criteria (table 1).

**Table 3:** Summary of published sensitivity and specificity estimates of several molecular methods techniques for HAT.

Authors	Participant countries	Test	Target	Number of participant (case/controls)	Sensitivity (CI95%)	Specificity (CI95%)	Remarks
Kabiri et al[^55^]	G-E&Angola	PCR	ESAG6/7	23/36	86•9[65•3–96•5]	97•2[83•8–99•8]	
Penchenier et al[^57^]	Cameroon,G-E,RCA	PCR	Satellite DNA	155/1654	99•4[96–100]	96•8[95•8–97•6]	
Kyambadde et al[^79^]	Uganda	PCR	Satellite DNA	14/21	100[73•2–99•3]	71•4[47•7–87•8]	Comparison of PCR results, CATT and HCT
Solano et al[^70^]	IC	PCR	Satellite DNA	26/49	100[84•0–99•6]	91•8[79•5–97•3]	
Radwanska et al[^54^]	IC	PCR	TgsGP gene	14/78	100[73•2–99•3]	100[91•3–99•8]	
Becker et al[^59^]	Sudan	Real-time PCR	Satellite DNA	13/5	100[71•6–99•3]	100[46•3–98•1]	
Koffi et al[^80^]	IC	PCR	Satellite DNA	38/463	100[88•6–99•8]	89•8[86•6–92•3]	
Deborggraeve et al[^68^]	DRC	PCR-OC	18S rDNA	26/47	100[84•0–99•6]	100[90•6–99•8]	
Njiru et al[^61^]	Uganda	LAMP	RIME	8/12	75•0[35•6–95•5]	100[70•0–99•2]	Archived samples
Mugasa et al[^66^]	Uganda & DRC	NASBA-RT	18S rRNA	33/50	93•9[78•3–98•9]	100[91•1–99•8]	Controls: nonendemic & endemic controls
Mugasa et al[^67^]	Uganda & DRC	NASBA-OC	18S rRNA	36/27	97•2[83•8–99•9]	59•3[39•0–77•0]	
Matovu et al[^65^]	Uganda & DRC	NASBA-OC	18S rRNA	68/122	97•1[90•0–99•2]	99•2[95•6–99•9]	
Matovu et al[^65^]	Uganda & DRC	PCR-OC	18S rDNA	68/123	82•4[71•6–89•6]	99•2[95•5–99•9]	
Deborggraeve et al[^58^]	DRC	PCR	18 rDNA	358/129	88•4[63•6–73•3]	99•2 [97•7–100]	

**Loop-mediated isothermal amplification (LAMP)** can amplify specific DNA sequences under a constant temperature, making it more feasible in less equipped laboratories [61]. LAMP requires 2 specific inner and outer primers and is based on autocycling strand displacement DNA synthesis by Bst DNA polymerase. A trypanozoon-specific LAMP assay based on repetitive insertion mobile element (RIME) was tested on samples [61]. LAMP is an amplification method able to detect a single nucleotide difference [71], [72]. LAMP is carried out at a constant temperature (usually in the range of 60–65*°*C) which eliminates the need of thermal cycler and shortens the reaction time by eliminating time lost during thermal changes. In 35 minutes, using a simple water bath, LAMP assay is able to detect both *T. b. gambiense* and *T. b. rhodesiense* directly from blood, serum or CSF samples. LAMP has been evaluated on small numbers of samples only. Sensitivity was 75% based on only 8 archived samples [61] and specificity based on 12 samples was 100% (table 3). LAMP is easier to perform than conventional PCR methods which require purified DNA. The sample is added to a microcentrifuge tube and mixed with primers, substrates, and a DNA polymerase capable of strand displacement. Monitoring of DNA amplification can be done with naked eye by observing either turbidity or fluorescence [71]. The future adoption of LAMP as a diagnostic tool for *Trypanosoma* infections in rural endemic regions shows promise but further validation studies are needed. When compared to classical PCR techniques, LAMP performs better in terms of user friendliness but has the same problems as PCR in relation to the other ASSURED criteria (table 1).

#### NASBA

Real-time nucleic sequence based amplification is a RNA amplification. A total volume of 10 µl of reaction mixture containing KCl and primers is incubated with RNA extract and control RNA in presence of molecular beacon at 65°C for 2 min. The reaction is subsequently cooled to 41°C for 2 min before adding enzyme mixture from a basic kit to each reaction mixture. The addition of enzyme starts the isothermal amplification at 41°C, which is continued for 90 min. The signal produced by the negative control sample is automatically subtracted from that of the analytical samples. The number of parasites is calculated from time to positivity, that is, the time point at which emitted fluorescence exceeds the baseline [66]. The sensitivity of NASBA, in a small sample was estimated at 69·5% and specificity at 100% [66]. The test needs to be further evaluated but will certainly be problematic in terms of affordability, user friendliness, robustness, and being equipment-free (table 1).

#### Oligochromatography (OC)

In brief, DNA or RNA is amplified by PCR or NASBA [65], [67], [68] after which the amplification products are detected by dipstick. Dipstick test results are read after 10 minutes. PCR-OC and NASBA-OC have a sensitivity ranging from 73·0% to 97·1% and specificity that ranges from 99·2% to 100% (table 3). Just like the other molecular methods, the test does not match the ASSURED criteria for affordability, user friendliness, robustness, and being equipment-free (table 1).

## Discussion

Our literature search yielded 46 articles reporting on five antibody detection tests, five conventional parasitological confirmation tests, and three molecular methods. The antibody detection tests are primarily screening tests; they lack adequate specificity to be used as diagnostic tests. Only the thermostable format of the CATT (CATT-D10) appears a realistic option for use at the health center level. Some concern is raised about its format, as it is not an individual test. Once a 10-unit vial has been opened it has to be used the same day. Given the generally low attendance rates at health centers in many sub-Saharan African countries, wastage is inevitable.

The other tests require a cold chain (classic CATT) or sophisticated equipment and well trained personnel (CATT on diluted samples, Latex*/T.b. gambiense*, IFAT, ELISA and immune trypanolysis). The fact that all these tests can be performed on dried capillary blood samples collected on filter paper does offer some perspective for their use in surveillance systems in which samples are collected at health centers but processed at a more central laboratory. New lateral flow tests are currently being developed for antibody detection in HAT [73]. Such tests will not require equipment, are thermostable and are marketed in individual formats. If they become available at an affordable price and are sufficiently sensitive and specific, they will fulfil the ASSURED criteria.

The parasitological confirmation tests, wet blood film, lymph node aspirate and thick blood film are feasible at health center level. They all do require a microscope but are easy to perform. The main problem is their lack of sensitivity, even if combined [26]. Any person testing positive on a serological screening test but negative on a combination of these three parasitological confirmation methods would have to be referred to a higher level of the health system for testing with more sensitive methods. The parasitological confirmation tests using a concentration step, mHCT, mAECT and QBC all require centrifugation and therefore electricity; they are also more complex to perform which limits their potential for use at health center level.

The molecular methods, PCR, LAMP, NASBA and NASBA-OC all require sophisticated equipment and electricity. PCR requires an expensive thermocycler and highly trained personnel. NASBA systems and LAMP do not require a thermocycler. If their close to 100% specificity is confirmed, they could be used as diagnostic confirmation tests at central laboratories. However, in a recent review, Wastling and Welburn [74] highlight concerns about LAMP as a clinical diagnostic tool for use in remote settings, citing the impracticality of the requirements for template preparation, need of heating blocks, electricity, a cold chain, and additional equipment not available in kit format. For LAMP to be feasible at health center level for HAT diagnosis, the format needs to be further improved as was done for a LAMP for Tuberculosis which uses a lyophilised reaction mix that includes Bst polymerase, avoiding the need for a cold chain [75]. Moreover, diagnostic accuracy still needs to undergo further validation in clinical context, in large prospective studies.

Even if based on a positive CATT-D10 followed by a positive thick blood film or lymph node aspirate a certain proportion of HAT cases can be diagnosed at health center level, they would still need to be referred to the district hospital to determine the stage of the disease. This requires a lumbar puncture which is a procedure best confined to higher level facilities. Treatment for stage 2 HAT patients is currently also very complex, requiring two intravenous infusions daily for one week as well as an oral drug three times daily for 10 days [76]. For all these reasons, with the possible exception of a few dedicated centers in highly endemic districts, the role of the primary care health center in HAT diagnosis will probably be limited to screening and referral. For confirmation purposes mAECT and mHCT are for the time being the best candidates although their use is also confined to district hospital level.

If further simplified LAMP and/or NASBA-OC may become an option as diagnostic confirmation tests.

The advent of a simple light-emitting diode (LED)-based fluorescence microscope using acridine orange may offer new opportunities for diagnosis of HAT at health center level. Nanobody based parasite antigen detection systems being developed may allow parasite detection by fluorescence microscopy [77]. Further evaluation studies are needed to properly assess the potential for field use of these new methods.

Our literature review has some limitations. Articles in other languages than English and French were not included and the search was limited to the PubMed database. Only ten out of the 46 articles retained respected the Standards for Reporting of Diagnostic Accuracy (STARD) check list for quality of reporting of diagnostic accuracy studies [78]. This often made the comparison between studies difficult because of variations in design [78]. Often, the sensitivity and specificity were calculated on a non-representative sample.

## Conclusion

The most suitable test for screening for HAT at health center level available today is the CATT-D10, though it might in the near future be replaced by a newly developed lateral flow test. Some of the classical parasite detection tests are feasible at health center level for case confirmation but they cannot be relied upon to exclude the diagnosis of HAT. Under the present circumstances the role of primary care health centers in HAT in endemic areas therefore seems limited to screening and referral of patients with suspected HAT to a more central level because the available diagnostic confirmation tests either lack sensitivity or are too demanding in terms of technical skills and/or equipment. In an era of dwindling resources and diminishing action radius of mobile teams HAT control programs need to seriously rethink their approach to case detection and surveillance.

## Supporting Information

Appendix S1 Search terms in MEDLINE.
(DOC)

Checklist S1 Prisma 2009 checklist for systematic reviews and meta-analyses
(DOC)

Flowchart S1 (DOC)

Text S1 (DOCX)
