# Supplementary material for: Human African Trypanosomiasis Diagnosis in First-Line Health Services of Endemic Countries, a Systematic Review
Source: PLoS Negl Trop Dis. 2012 Nov 29;6(11):e1919. doi: 10.1371/journal.pntd.0001919 (PMC3510092; doi:10.1371/journal.pntd.0001919)
Supplement: Appendix S1 — Search terms in MEDLINE. (DOC) [file pntd.0001919.s001.doc]

**Appendix S1:** Search terms in MEDLINE .

"Trypanosomiasis, African/diagnosis"[Mesh] AND (Humans [restriction] language: English OR French AND ("1970/01/01"[PDat] : "2011/12/31"[PDat]))
