# Supplementary material for: Human African Trypanosomiasis Diagnosis in First-Line Health Services of Endemic Countries, a Systematic Review
Source: PLoS Negl Trop Dis. 2012 Nov 29;6(11):e1919. doi: 10.1371/journal.pntd.0001919 (PMC3510092; doi:10.1371/journal.pntd.0001919)
Supplement: Flowchart S1 — (DOC) [file pntd.0001919.s003.doc]

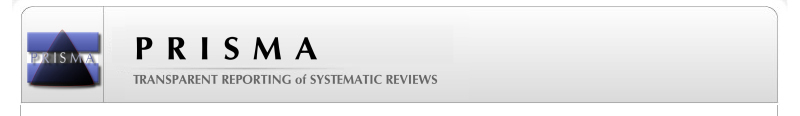
**PRISMA 2009 Flow Diagram**

**Screening**

**Included**

**Eligibility**

**Identification**

Records identified through database searching
(n =428 )

titles and abstracts were further reviewed
(n =357 )

Reviews excluded
(n =71 )

Full-text articles and abstracts were further reviewed for inclusion and exclusion criteria

(n= 62)

(n =62 )

Full-text articles excluded, because dealing with other parasites
(n = 16 )

Full-text were included in systematic review
(n = 46 )

Full-text articles excluded editorials, viewpoints, case reports, clinical drug trials, biomarkers, qualitative studies, evaluation of HAT control programmes, cost-effectiveness studies, studies on animal trypanosomiasis)
 (n =295 )
