# Supplementary material for: Human African Trypanosomiasis Diagnosis in First-Line Health Services of Endemic Countries, a Systematic Review
Source: PLoS Negl Trop Dis. 2012 Nov 29;6(11):e1919. doi: 10.1371/journal.pntd.0001919 (PMC3510092; doi:10.1371/journal.pntd.0001919)
Supplement: Text S1 — (DOCX) [file pntd.0001919.s004.docx]

**Summary of published sensitivity and specificity estimates of several serological HAT**

| Authors | Participant countries | Test | Antigen | Number of cases  and controls | Sensitivity  (CI 95%) | Specificity  (CI 95%) | Remarks |
| --- | --- | --- | --- | --- | --- | --- | --- |
| Pepin et al^[23]^ | DRC(ex:Zaïre) | CATT | Litat 1.3 | 47/4009 | 100[90∙6–99∙8] | 88∙3[87∙3–89∙3] | Active cases finding  and included consecutively |
| Pepin et al^[23]^ | DRC | CATT | Litat 1.3 | 189/456 | 98∙9[95∙8–99∙8] | 78∙3[741–82∙0] | Passive case finding |
| Bafort et al^[24]^ | Republic of South Africa | CATT | Litat 1.3 | 0/242 | NR | 97∙1[93∙9–98∙7] | All subjects included  are controls |
| Noireau et al^[34]^ | Congo | CATT | Litat 1.3 | 96/3434 | 68∙8[58∙4–77∙6] | 94∙8[93∙7–95∙2] |  |
| Noireau et al^[16]^ | Congo | CATT | Litat 1.3 | 52/118 | 100[91∙4–99∙8] | NR |  |
| Miezan et al^[15]^ | Ivory Coast(IC) | CATT | Litat 1.3 | 56/397 | 100[92∙0 –99∙8] | 98,3[96∙3–99∙3] | |
| Miezan et al^[15]^ | IC | CATT | Litat 1.3 | 16/1618 | 100[75∙9–99∙4] | 98∙6[97∙9–99∙1] |  |
| Jamonneau et al^[25]^ | IC | CATT | Litat 1.3 | 0/425 | NR | 92∙5[89∙5 –94 ∙7] | Random inclusion |
| Magnus et al^[21]^ | Belgium | CATT | Litat 1.3 | 0/358 | NR | 97.8[95.6–99.0] | Non endemic controls |
| Magnus et al^[21]^ | Uganda, DRC, IC | CATT | Litat 1.3 | 115/1854 | 90∙4 [83∙5–95∙1] | 96.5 [95.6–97.3] |  |
| Magnus et al^[21]^ | Uganda, DRC, Guinée Equatoriale(G-E) | CATT | Litat 1.3 | 12/227 | 100 [73∙5–100] | 90.3[85.7–93.8] |  |
| Magnus et al^[21]^ | G-E | CATT | Litat 1.3 | 11/89 | 90∙9[58∙7–99∙8] | 95.5 [88.9–98.8] |  |
| Truc et al^[17]^ | République Centrafricaine(RCA) | CATT | Litat 1.3 | 56/484 | 98∙2[89∙2–99∙9] | 95∙2[92∙8–96∙9] |  |
| Truc et al^[17]^ | IC | CATT | Litat 1.3 | 10/390 | 100[65∙5–99∙1] | 96∙7[94∙3–98∙2] |  |
| Penchenier et al^[22]^ | Cameroon | CATT | Litat 1.3 | 16/5239 | 98∙7[74∙1–99∙8] | 91∙8[91∙0–92∙5] |  |
| Penchenier et al^[22]^ | RCA | CATT | Litat 1.3 | 59/2019 | 96∙4[87–99∙3] | 95∙2[94∙1 –96∙1] |  |
| Inojosa et al^[19]^ | Angola | CATT | Litat 1.3 | 10/14436 | 100[65∙5–99∙1] | 98∙3[98∙1–98∙5] | Active case finding and  included consecutively |
| Inojosa et al^[19]^ | Angola | CATT | Litat 1.3 | 34/345 | 100[87∙4 –99∙7] | 85∙8[81∙6–89∙2] | Passive cases finding |
| Lejon et al^[20]^ | DRC, Benin | CATT | Litat 1.3 | 78/128 | 100[94∙1–99∙9] | 85∙2[77∙6 –90∙6] |  |
| El rayah et al^[13]^ | Sudan | CATT | Litat 1.3 | 3/1378 | 100[31∙0–96∙8] | 96∙3[95∙1–97∙2] |  |
| Lutumba et al^[26]^ | DRC | CATT on sample dilution | Litat 1.3 | 154/282 | 78∙8[71∙2–85∙1] | 58∙5[52∙3–64∙5] |  |
| Elrayah et al^[13]^ | Sudan | CATT on sample dilution | Litat 1.3 | 0/203 | NR | 99∙5[97∙3–100] |  |
| Miezan et al^[15]^ | IC | CATT on filter paper | Litat 1.3 | 56/397 | 100[92∙0–99∙8] | 98∙2[96∙2–99∙2] |  |
| Miezan et al^[15]^ | IC | CATT on filter paper | Litat 1.3 | 16/1698 | 100[75∙9–99∙4] | 99∙4[98∙9–99∙7] |  |
| Noireau et al^[16]^ | Congo | CATT on filter paper | Litat 1.3 | 52/118 | 94∙2[83∙0–98∙5] | 100[96∙1–99∙9] |  |
| Laveissière et al^[29]^ | IC | CATT on filter paper | Litat 1.3 | 0/24 344 | NR | 98∙5[98∙3–98∙6] |  |
| Truc et al^[17]^ | RCA | CATT on filter paper | Litat 1.3 | 56/484 | 89∙3[77∙5–95∙6] | 93∙4[90∙7–95∙4] |  |
| Truc et al^[17]^ | IC | CATT on filter paper | Litat 1.3 | 10/390 | 90[54∙1–99∙5] | 98∙2[96∙2–99∙2] |  |
| Chappuis et al^[12]^ | Sudan | CATT on filter paper | Litat 1.3 | 99/0 | 90∙9[83∙0–95∙5] | NR |  |
| El rayah et al^[13]^ | Sudan | CATT on filter paper | Litat 1.3 | 0/203 | NR | 100[98∙5 –100] |  |
| Hasker et al^[14]^ | DRC | CATT on filter paper | Litat 1.3 | 100/3212 | 92∙7[87∙4–98∙0] | 99∙4[99∙0–99∙9] |  |
| Wery et al^[35]^ | DRC | IFAT | *T.b.brucei* | 121/1147 | 99∙2 [94∙9–100] | 99∙4[98∙7–99,7] |  |
| WHO et al^[31]^ | Congo, DRC, Uganda, Nigeria | IFAT | *T.b.gambiense* | 47/11 | 87∙2 [73∙5–94∙7] | 100[67∙9–99∙2] |  |
| Frezzil et al^[33]^ | Congo | IFAT | *T.b.gambiense* | 200/165 | 99∙0[96∙1–99∙8] | 100[97∙2–99∙9] |  |
| Duvallet et al^[32]^ | IC | IFAT | *T.b.gambiense* | 13/1431 | 100 [71∙7–99∙3] | 98,3[97∙5–98∙9] |  |
| Noireau et al^[34]^ | Congo | IFAT | *T.b.gambiense* | 95/255 | 94∙7[87∙5–98∙0] | 100 [98∙1–100] |  |
| Büscher *et* al^[30]^ | Congo, DRC, Uganda, Nigeria, IC,G-E, Gabon, Sudan | Latex | Litat1.3,1.5,1.6 VSG | 240/173 | 97∙2[94∙0– 98∙8] | 91[85∙5–94∙6] | Dilution 1/8 |
| Jamonneau et al^[25]^ | IC | Latex | Litat1.3,1.5,1.6 VSG | 0/425 | NR | 98∙1 [96∙2–99∙1] | Dilution 1/4 |
| Truc *et* al^[17]^ | IC | Latex | Litat1.3,1.5,1.6 VSG | 54/484 | 90[78∙3–96∙0] | 99∙4[98∙1–99∙8] |  |
| Truc et al^[17]^ | RCA | Latex | Litat1.3,1.5,1.6 VSG | 10/390 | 67∙9[33∙6–90∙8] | 99∙0[97∙2 –99∙7] |  |
| Penchenier et al^[22]^ | Cameroon | Latex | Litat1.3,1.5,1.6 VSG | 16/5239 | 100 [75∙9–99∙4] | 97∙6 [97∙1–98∙0] | Dilution 1/4 |
| Penchenier et al^[22]^ | RCA | Latex | Litat1.3,1.5,1.6 VSG | 59/2019 | 100 [92∙4–99∙8] | 96∙1 [95∙1–96∙9] | Dilution 1/4 |
| El rayah *et* al^[13]^ | Sudan | Latex | Litat1.3,1.5,1.6 VSG | 0/203 | NR | 99∙0 [96∙5–99∙9] | 1/8 was the cutt-of  dilution |
| Meirvenne et al^[38]^ | Congo,DRC, Uganda, Nigeria, IC, G-E, Gabon, Sudan | Trypanolysis | *T.b.gambiense* VAT clones LiTAT 1.1 to 1.10,VAT clones AnTaT 11.17 to 11.20 | 340/267 | 98∙8[96∙8–99∙6] | 100[98∙2–100] |  |
| Lutumba et al^[26]^ | DRC | Trypanolysis | *T.b.gambiense* VAT clones LiTAT 1.1 to 1.10,VAT clones AnTaT 11.17 to 11.20 | 151/227 | 97∙2[92∙9–99∙2] |  |  |
| Jamonneau et al^[40]^ | G-E,IC | Trypanolysis | Cloned *T.b. gambiense* VATs LiTat1.3, LiTat 1.5  and LiTat 1.6 | 71/0 | 100[93∙6–99∙9] | NR |  |
| Lejon et al^[36]^ | DRC | ELISA | Litat1.3,Litat1.5,Litat1.6 VSG | 23/14 | 100[85∙2–100] | 100[76∙8–100] | ELISA on saliva |
| Lejon et al^[20]^ | Bénin,DRC | ELISA | Litat1.3,Litat1.5,Litat1.6 VSG | 78/128 | 96∙3[88∙6– 99∙1] | 94∙7[88∙9–97∙7] | ELISA on saliva |
| Lejon et al^[20]^ | Bénin,DRC | ELISA | Litat1.3,Litat1.5,Litat1.6 VSG | 78/128 | 98∙7[92∙1–99∙9] | 98,4[93∙8–99∙7] | ELISA on serum |
| Elrayah et al^[13]^ | Sudan | ELISA | Litat1.3,Litat1.5,Litat1.6 VSG | 0/200 | NR | 98∙5[95∙4–99∙6] | ELISA on plasma |
| Elrayah et al^[13]^ | Sudan | ELISA | Litat1.3,Litat1.5,Litat1.6 VSG | 0/196 | NR | 98∙5[95∙6– 99∙7] | ELISA on filter paper |
| Hasker et al^[14]^ | DRC | ELISA | Litat1.3,Litat1.5,Litat1.6 VSG | 100/3212 | 82∙8[75∙3–90∙4] | 99∙8[99∙5–100] | ELISA on filter paper |
